# Characterizing the Anti-HIV Activity of Papuamide A

**DOI:** 10.3390/md20080027

**Published:** 2008-10-08

**Authors:** Cynthia D Andjelic, Vicente Planelles, Louis R Barrows

**Affiliations:** 1 University of Utah, Department of Pharmacology and Toxicology, 30 S. 2000 E. Rm.201, Salt Lake City UT 84112, USA Tel. +1-801-581-4547; Fax: +1-801-585-9347; E-mail: C.D.Carlson@utah.edu; 2 University of Utah, Department of Pathology, Salt Lake City, UT USA E-mail: vicente.planelles@path.utah.edu

**Keywords:** HIV, papuamide A, cyclic depsipeptide, entry inhibitor, marine metabolite

## Abstract

Papuamide A is representative of a class of marine derived cyclic depsipeptides, reported to have cytoprotective activity against HIV-1 *in vitro*. We show here that papuamide A acts as an entry inhibitor, preventing human immunodeficiency virus infection of host cells and that this inhibition is not specific to R5 or X4 tropic virus. This inhibition of viral entry was determined to not be due to papuamide A binding to CD4 or HIV gp120, the two proteins involved in the cell-virus recognition and binding. Furthermore, papuamide A was able to inhibit HIV pseudotype viruses expressing envelope glycoproteins from vesicular stomatitis virus or amphotropic murine leukemia virus indicating the mechanism of viral entry inhibition is not HIV-1 envelope glycoprotein specific. Time delayed addition studies with the pseudotyped viruses show that papuamide A inhibits viral infection only at the initial stage of the viral life cycle. Additionally, pretreatment studies revealed that the virus, and not the cell, is the target of papuamide A’s action. Together, these results suggest a direct virucidal mechanism of HIV-1 inhibition by papuamide A. We also demonstrate here that the other papuamides (B-D) are able to inhibit viral entry indicating that the free amino moiety of 2,3-diaminobutanoic acid residue is not required for the virucidal activity.

## 1. Introduction

**Figure 1.**
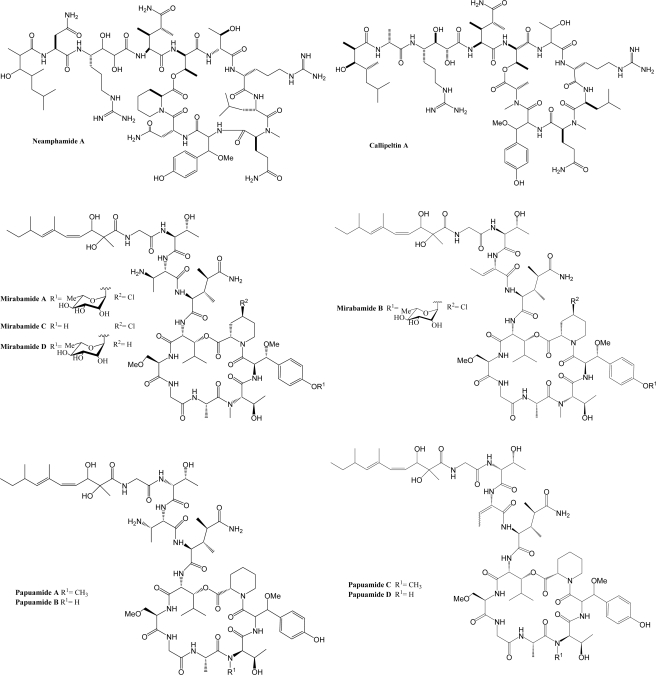
Chemical structures of neamphamide A, callipeltin A, mirabamides A–D and papuamides A–D.

Marine organisms have proven to be an excellent source of biologically active compounds against human immunodeficiency virus (HIV) [1, 2]. One class of molecules with potent cytoprotective activity consists of structurally similar cyclic depsipeptides isolated from sponges. These marine metabolites exhibit potent inhibition of HIV induced T cell death *in vitro* with selectivity indexes (SI, effective dose divided by the cytotoxic dose) ranging from 3 to 29. Included in this group are: neamphamide A (SI=9.29) [3, 4], callipeltin A (SI=29) [4, 5], mirabamides A–D (SI ranging from 3–13) [6] and papuamide A and B (SI=21 for both) [4, 7]. (Chemical structures provided in Fig. 1.) The mirabamides are the most recently discovered of this group and have been reported to inhibit HIV-1 envelope mediated cell-cell fusion in a recombinant vaccinia virus fusion assay [6].

Problems associated with current therapeutic treatments and the emergence of drug resistant HIV strains [8] establishes the need for new therapeutic treatments. Agents which inhibit viral entry are particularly sought after because they can prevent infection. HIV entry is often described as a three stage process: attachment of the virus to the target cell, co-receptor binding, and fusion of the virus and cell membranes. Virus attachment is generally through HIV gp120 recognition and binding to the host cell receptor, CD4 [9, 10]. Once the virus is attached to the cell, gp120 will undergo conformational changes exposing a new epitope which then interacts with a cellular chemokine co-receptor [11–13], typically either CCR5 or CXCR4. After this dual receptor interaction has taken place, gp120 and gp41 will undergo further conformational changes exposing the extracellular region of gp41[12–16]. The N-terminal region of gp41 contains what has been called a fusion peptide that inserts itself into the cell membrane [17]. Two helical regions of gp41 fold upon each other, bringing the virus and cellular membranes into close proximity. The resultant free energy change from this structural rearrangement is thought to be sufficient for lipid mixing and fusion of the two membranes to occur, thus allowing the viral core to enter the host cell [18–20]. Compounds that inhibit any stage of this entry process may not only be useful for the treatment of HIV/AIDS infected individuals, but also have the potential to be used as microbicides in the absence of a vaccine.

The structural uniqueness of natural product metabolites often leads to the identification of new targets in the treatment of diseases and novel inhibitors of viral entry are a much desired class of drugs. Therefore, we investigated the mechanism by which papuamide A protects against HIV induced cytopathicity. In the present study, papuamide A’s ability to inhibit HIV entry is demonstrated. Papuamide A is shown to target the virus and this virucidal mechanism is investigated. In addition, papuamides B, C and D are shown to also inhibit HIV entry.

## 2. Results

### 2.1 Inhibition of viral entry by papuamide A.

Investigation of papuamide A’s ability to inhibit HIV induced T cell death lead to the testing of papuamide A’s ability to inhibit HIV entry in the virion based fusion assay developed by Cavrois *et al.* [21]. This assay quantifies viral entry using the chimeric protein BlaM-vpr, β-lactamase linked to vpr, which is readily incorporated into virions produced by co-transfection with proviral DNA. Initial analysis was performed using an X4 tropic virus (virus that infects cells expressing CXCR4), produced by co-transfection with HIV-1_NL4-3_ proviral DNA, to infect HeLa T4+ cells expressing CXCR4. Fig. 2a shows representative microscope images of this assay for control cells (non-infected cells fluoresce green), HIV infected cells (infected cells fluoresce blue) and infected cells treated with papuamide A (showing a decrease in the presence of infected cells due to inhibition of viral entry). Fig. 2b graphs the results from this experiment and shows that papuamide A at a concentration of 710 nM inhibited approximately 80% of viral entry. Controls used included C34 (a fusion inhibitor) which effectively blocked viral entry and AZT (a reverse transcriptase inhibitor) which did not prevent viral entry as expected. To determine if this activity was X4 tropic specific, the ability of papuamide A to inhibit a R5 tropic virus (virus that infects cells expressing CCR5) was evaluated using HIV-1_NL(AD8)_, to infect the TZM-bl cells, HeLa T4+ cell line expressing CCR5. Papuamide A effectively inhibited the entry of the R5 tropic virus in a dose dependant manner (Fig. 2c). The dose response curve provides an EC_50_ (50% effective concentration) of approximately 114 nM (Fig. 2c). This potent effective concentration is similar to the EC_50_ of 71 nM obtained for papuamide A protection against HIV induced cell death determined using the cytoprotection 3-(4,5-dimethylthiazol-2-yl)-2,5diphenltetrazolium bromide (MTT) based assay of Kiser and colleagues [22] established in our laboratory (data not shown).

### 2.2 Papuamide A does not show significant binding to either sCD4 or gp120.

**Figure 3.**
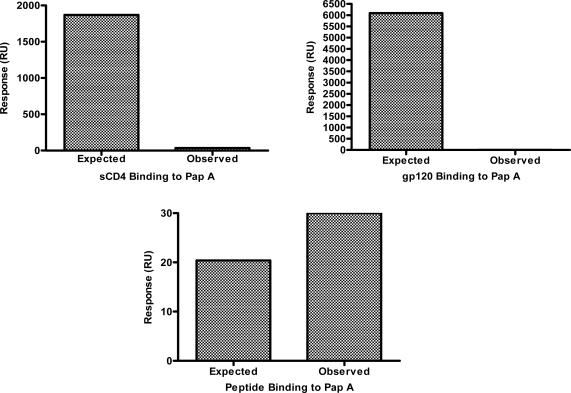
Surface plasmon resonance showed papuamide A did not interact with sCD4 and gp120. Graphs show the calculated expected binding response versus the observed binding response for papuamide A (Pap A) with sCD4, gp120 or positive control peptide binding.

The lack of tropism specificity demonstrated above suggested that the chemokine co-receptors (CXCR4 and CCR5) and the co-receptor binding domain of gp120 are not the targets of papuamide A inhibition. However, the initial step of HIV entry, binding of gp120 to CD4, is thought to be similar regardless of viral tropism, therefore, the ability of papuamide A to interact with CD4 or gp120 was assessed. Successful biotinylation of papuamide A without a significant loss of activity was achieved (data not shown) and the biotinylated papuamide A was utilized to quantify binding of sCD4 or gp120 by surface plasmon resonance (SPR). Fig. 3 shows the observed binding response (response in RU measured by the instrument) versus the expected binding response (see formula and explanation below) for sCD4 and gp120 with papuamide A. SPR binding responses for both proteins were determined not to be significant because the observed response was 600 times lower than the calculated expected response for sCD4 at the maximum concentration tested, and the observed response for gp120 was 54 times lower than the calculated expected response at the maximum concentration tested. Papuamide A was shown to interact with a positive control binding peptide, as expected in this system.

Values used to determine the expected response are in Table 1. The expected response at saturation is determined by the following formula: $\frac{LCR}{MWL}\times MWA$, (LCR=Ligand capture response, amount of papuamide A captured onto the biosensor chip; MWL=molecular weight of ligand, papuamide A; and MWA=molecular weight of analyte, the protein or peptide.)[23–26] The observed response, determined by the BIAcore instrument detector in response units (RU), can be lower than the expected response if the concentration of analyte used is below the dissociation constant (K_D_) concentration value, K_D_ values were calculated by the software from the binding responses obtained with multiple concentrations of analyte. When this is the case, the fold difference in concentration values (K_D_/concentration of analyte used) is approximately proportionate to the fold difference in responses (expected response/observe response). Therefore, to correct for the expected response at the maximum concentration tested, the expected response at saturation was divided by the fold difference in concentration values to determine the expected response at the maximum value tested.

### 2.3 Papuamide A does not exhibit virus envelope specificity.

The proteins known to be critical for the initial stage of virus attachment to the cell do not appear to be the targets of papuamide A. This finding lead to the investigation of a more general mechanism of viral entry inhibition. Inhibition of infection was evaluated using multiple pseudotype viruses (engineered HIV virions bearing various envelope glycoproteins). CEM-SS, a human T4 lymphoblastoid cell line, was used for all infections except for those using pseudotype virus expressing JRFL, which requires the presence of CCR5. For the JRFL pseudotype virus experiments, CEM.NKR-CCR5, a CEM derived cell line expressing CCR5, was used. Pseudotype viruses were produced by co-transfection of envelope glycoprotein plasmid and an env-defective HIV vector, DHIV-3-GFP, [27] which contains a green fluorescence protein (GFP) reporter gene in place of *nef* (this allows for quantification of HIV infection by flow cytometry). Papuamide A was tested at a concentration of 174nM which was previously determined to inhibit approximately 50% of infection in this system (data not shown).

**Figure 4.**
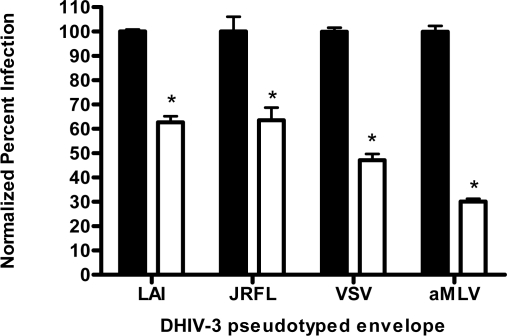
Papuamide A inhibits infection by pseudotype viruses presenting LAI (X4 tropic), JRFL (R5 tropic), vesicular stomatitis virus (VSV) or amphotropic murine leukemia virus (aMLV) envelope glycoproteins. Black columns indicates no papuamide A added, white columns indicate infection in presence of 178 nM papuamide A. Data is graphed as the mean ± standard error, n≥3. Percent infection was significantly different (p < 0.05) between infected cells that were not treated and infected cells treated with papuamide A (*).

First, the ability of papuamide A to inhibit infection in this system was confirmed using viruses expressing LAI (X4 tropic) or JRFL (R5 tropic) envelope proteins (Fig. 4). This lack of viral tropism specificity was consistent with that observed in the virus entry inhibition studies above. Next, the defective HIV-GFP virus was pseudotyped with envelope glycoproteins from either vesicular stomatitis virus (VSV) or amphotropic murine leukemia virus (aMLV), and the sensitivity to papuamide A inhibition was tested. Papuamide A effectively inhibited infection by these pseudotype viruses (Fig. 4). These results confirm that papuamide A does not act by inhibiting the recognition and attachment of the virus to the cell and also suggests that papuamide A’s activity in not selective for gp41 mediated fusion. VSV and aMLV envelopes result in the virus entering through a receptor mediated endocytic pathway [27–30] and through the PIT-2 sodium phosphate co-transporter mediated pathway [31, 32], respectively.

The pseudotype virus assay described above is a single round infectivity assay that can detect inhibition of early viral life cycle events. To confirm the activity of papuamide A in the pseudotyped virus system was due to inhibition of the initial viral entry steps, time delayed addition studies were performed. Compounds which are active during the entry process of the viral life cycle show a strong time dependency in their effectiveness. This is illustrated in Fig. 5a with the controls C34, an inhibitor of fusion, and AZT, a reverse transcriptase inhibitor. C34 showed a significant decrease in effectiveness when addition was delayed by 1 hour, with an even greater loss of activity when addition was delayed 2 hours. The profile of AZT showed no difference in activity when added 1 hour after infection, and only a minor loss of activity when added 2 hours after infection. Papuamide A’s profile (Fig. 5b) was similar to that of C34, indicating the observed activity is at the initial stages of the viral life cycle. A similar loss of inhibition due to delayed addition was also observed for pseudotype virus expressing VSV envelope glycoprotein when compared to pseudotype virus expressing an HIV envelope glycoprotein (Fig. 5c).

### 2.4 Papuamide A interacts directly with the virus, not the target cell, to inhibit infection.

Current FDA approved inhibitors of viral entry and the majority of those under investigation target CD4, gp120, the chemokine co-receptors, or gp41 mediated fusion.[33] Papuamide A’s inhibition of viral entry has been shown to be independent of these proteins and appears to be novel in its mechanism. To further evaluate papuamide A’s mechanism, pretreatment studies were performed to determine if papuamide A was interacting directly with the virus or the target cell.

In the virion fusion assay, cells were incubated with papuamide A for 2 hours prior to infection. Papuamide A containing medium was removed followed by two washes. This pretreatment protocol did not result in inhibition of viral entry demonstrating that papuamide A does not have an irreversible effect on the cell (Fig. 6a). Preincubation of the cells with papuamide A overnight also failed to inhibit viral infection (data not shown). Modification of the fusion assay was performed so that HIV was pretreated with papuamide A for 30 minutes prior to infection. Virus pretreatment, followed by dilution of virus, resulted in a rate of inhibition similar to when papuamide A was added at the same time as virus (Fig. 6a). However, in this experiment papuamide A could only be diluted, not completely removed from the virus and medium. Effective removal of unbound papuamide A after virus incubation requires ultracentrifugation, but ultracentrifugation of HIV enveloped virus greatly decreases virus infectivity [34, 35]. To overcome this problem, VSV-G pseudotype virus was used for pretreatment studies because VSV-G pseudotype virus has been shown to retain infectivity after ultracentrifugation [34]. VSV-G pseudotype virus was treated with papuamide A and incubated overnight. Virus was pelleted, papuamide A containing supernatant removed and pellet suspended in fresh medium. Virus pretreatment with papuamide A resulted in an approximate 50% decreased infectivity of the virus (Fig. 6b). This decreased infectivity was the same as that observed when papuamide A was added to cells at the same time as VSV-G pseudotype virus (control virus underwent overnight incubation with vehicle and ultracentrifugation to control for potential loss of infectivity due to this process). These results indicate that papuamide A either remains stably bound in an inhibitory association with the virions after ultracentrifugation and washing or, alternatively, papuamide A inactivates the virus.

### 2.5 Effect of binding phosphatidylserine

Papuamide B only differs from papuamide A by one methyl group. Papuamide B has been shown to bind to and cause leakage of phosphatidylserine (PS) containing liposomes, but not those composed solely of phosphatidylcholine [36]. PS is a phospholipid normally present on the inner leaflet of the cell membrane, but during apoptosis the regulation of PS is altered and PS is present on the outer leaflet. Callahan *et al.* showed PS is present in the envelopes of HIV virions and reported that binding of PS by Annexin V, a specific binder of PS, was able to inhibit monocyte and macrophage infection, but not T cell infection [37]. These results suggest that binding of PS by papuamide A may disrupt the viral membrane. Therefore, the possibility of PS being the target of papuamide A’s virucidal activity was investigated.

Papuamide A’s ability to bind to phosphatidylserine was observed using surface plasmon resonance. Twenty percent phosphatidylserine and 80% phosphatidylcholine (PC) vesicles were tested alongside 100% PC vesicles. The PS:PC vesicles exhibited binding to papuamide A while the 100% PC vesicles did not (data not shown). This indicates there is a preferential binding of papuamide A to phosphatidylserine. To determine if binding of PS alone was sufficient to inhibit viral entry, annexin V (AV) was tested in the virion based fusion assay. AV (tested at the concentrations similar to and higher than that shown to inhibit HIV infection) did not have any inhibitory effect on either X4 or R5 virus entry, while papuamide A remained active (Fig. 7a). In addition the effect of papuamide A was unchanged in the presence of AV when tested in the pseudotype assay (Fig. 7b). The results suggest that PS binding does not appear to be necessary for papuamide A to block entry.

### 2.6 Papuamides B-D inhibit viral entry

**Figure 8.**
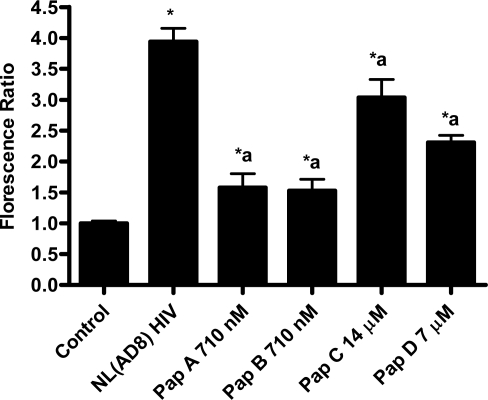
Papuamides B-D inhibit HIV entry. Papuamides A-D were tested at the stated concentrations against NL(AD8) virus in the virion based fusion assay. Control represents uninfected cells and NL(AD8) HIV represents untreated infected cells. Viral entry is reported as a fluorescence ratio calculated from the amount of blue versus green fluorescence, normalized to control. Results graphed as the mean ± standard error, n≥4. Fluorescence ratio was significantly different (p < 0.05) from control (*) or from untreated HIV infected cells (a).

While we were limited by the amount of papuamides B-D available for testing, the ability of these analogs to inhibit HIV entry was determined. Papuamide B exhibited similar inhibition of viral entry, approximately 80% inhibition at 710 nM, when compared to papuamide A (Fig. 8). Papuamides C and D were less potent, inhibiting approximately 30% and 55%, respectively, at concentrations of 40 and 20 fold higher (Fig. 8). Furthermore, papuamides C and D effective concentrations were closer to their cytotoxic concentrations suggesting a narrower “therapeutic” window for these analogs *in vitro* (data not shown).

## 3. Conclusion

In this study, we determine that the mechanism of papuamide A cytoprotection against HIV is through inhibition of virus entry. Papuamide A’s ability to inhibit both X4 and R5 tropic virus was similar to that recently published for the mirabamides, which were shown to inhibit fusion [6]. Further work shows that papuamide A inhibition does not target the key proteins involved in the viral entry process. Instead papuamide A works through a direct interaction with the virus and its virucidal activity appears to be independent of the type of envelope glycoprotein expressed. Phosphatidylserine (PS) a phospholipid present on the viral membrane has been proposed to be the target of papuamide B. Data presented here shows that while papuamide A does selectively bind PS, binding of PS alone is not sufficient to block viral entry.

Papuamide A’s activity may be representative of the group of marine depsipeptides shown to inhibit HIV induced cytopathicity. Papuamide A shares many chemical features with this group including an aliphatic tail, depsipeptide cyclization, a 3,4-dimethylglutamine residue and an available tyrosine hydroxyl (glycosylated in the mirabamides). Previously anti-HIV activity was reported for papuamides A and B. Here we show that papuamide C and D inhibit HIV entry as well, although less potently than A and B. This suggests that the free amino group of the 2,3-aminobutanoic acid residue of papuamides A and B is not required although it may contribute to the proposed virucidal activity of these compounds.

A proposed model for the mechanism of virucidal activity for these compounds can be based upon the membrane targeting mechanism proposed for a antifungal sterol dependent lipopeptide [38]. In this model, the lipopeptide has an aliphatic tail which inserts itself into the fungal membrane. The interaction is then stabilized through tyrosine binding to sterol present in the fungal membrane [38]. Cholesterol is a major component of the HIV viral membrane due to budding of the virus from cellular membrane microdomains rich in cholesterol and sphingolipids [39–41]. Common attributes of papuamide A and the other active depsipeptides include a tyrosine available to interact with cholesterol and a hydrophobic tail which could insert into the viral membrane. We hypothesize this could lead to disruption of the viral membrane resulting in a virucidal effect. Cooperative binding to PS may or may not contribute to papuamide A’s targeting of the viral membrane over the cellular membrane.

## 4. Experimental Section

### 4.1 Cells lines

The following cell lines were obtained through the AIDS Research and Reference Reagent Program: HeLa T4+, a human cervical epithelial carcinoma (HeLa) cell line rendered CD4+ by retrovirus-mediated gene transfer [42]; TZM-bl, a HeLa cell line stably expressing CD4 and CCR5 with β-galactosidase and luciferase reporter genes [43–45]; CEM-SS, a human T4 lymphoblastoid cell line [46, 47]; and CEM.NKR-CCR5, a human T4 lymphoblastoid cell expressing CCR5 [48, 49]. These cell lines were contributed by: Dr. Richard Axel; Dr. John C. Kappes, Dr. Xiaoyun Wu and Tranzyme Inc.; Dr. Peter L. Nara; and Dr. Alexandra Trkola, respectively. The HeLa T4+ and TZM-bl cell lines were maintained in Dulbecco’s modified Eagles medium (DMEM) supplemented with 10% fetal bovine serum (FBS) and the CEM-SS and CEM.NKR-CCR5 cell lines maintained in RPMI supplemented with 10% FBS and 200 μM L-glutamine. The human embryonic kidney (HEK) cell line transformed to express the large T antigen, 293FT (Invitrogen, Carlsbad, CA), was maintained in DMEM, 10% FBS and 200 μM L-glutamine.

### 4.2 Plasmids

The pAdVAntage plasmid was purchased through Invitrogen (Carlsbad, CA). Dr. Eric O. Freed at the National Institutes of Health (NIH) kindly provided pNL4-3 and pIIINL4env [50]. pNL(AD8) was obtained through the AIDS Research and Reference Reagent Program, contributed by Dr. Eric O. Freed [51]. The plasmid pSCA was kindly provided by Paula M. Cannon, University of Southern California [52]. The following plasmids have been described elsewhere, pMM310 [21], pDHIV-3 [27], pHCMV-VSVG [34], pLET-JRFL [53], and pLET-LAI [53, 54].

### 4.3 Drugs

Papuamides A-D were the generous gift of Drs. David Williams and Raymond Andersen from the University of British Columbia [7]. Papuamide A was dissolved in 50:50, H_2_O:MeOH at a stock concentration of 10 mg/ml. Azidothymidine (AZT) was purchased from Sigma-Aldrich (St. Louis, MO) and kept at a stock concentration of 10 mg/ml in DMSO. HIV-1 IIIB C34 Peptide (C34) was obtained through the AIDS Research and Reference Reagent Program contributed by the Division of AIDS, NIAID [55]. C34 was dissolved in H_2_O at a concentration of 1 mg/ml. All concentrations of drugs used in text and figures are provided as final concentration in culture.

### 4.4 Virion based fusion assay.

The fusion assay used was developed by Cavrois *et al.* [21]. Briefly, production of virus was accomplished by co-transfection of 20 μg of pMM310 (plasmid encoding β-lactamase linked to Vpr, BlaM-Vpr) [56],10 μg pAdVAntage (Invitrogen, Carlsbad, CA); and 60 μg pNL4-3(X4 tropic proviral DNA) [50] or pNL(AD8) (R5 tropic proviral DNA) [51] into 293FT cells using standard calcium phosphate transfection. Virus was collected after 48 hours and titered using the RETROtek p24 ELISA kit (ZeptoMatrix, Buffalo, NY). HeLaT4 or TZM-bl cells, 200μL at a concentration of 1 X 10^5^ cells/mL, were plated into 96 well, black walled, flat bottom plates and placed in a 37°C, 5% CO_2_ humidified incubator overnight for attachment. Medium was aspirated and 200 μL of medium containing virus, at a predetermined p24 concentration of 200 ng/mL, was added with test compounds or vehicle. Cells were incubated at 37°C, 5% CO_2_ for 2 hours. Virus was aspirated off and cells rinsed twice with room temperature CO_2_ independent medium (Invitrogen, Carlsbad, CA). Cells were then loaded with CCF2-AM fluorescent substrate (Invitrogen, Carlsbad, CA) according to manufacturer’s protocol. Cells were incubated, protected from light, for 1 hour at room temperature and washed twice with room temperature CO_2_ independent medium. Next, 200 μL of room temperature CO_2_ independent medium supplemented with 10% FBS and 2.5mM probenecid (Sigma-Aldrich, St. Louis, MO) was added and the reaction was allowed to develop for 5–7 hours.

When fusion occurs, β-lactamase (BlaM) present in the virus cleaves the CCF2-AM substrate. This cleavage results in a change of the fluorescence emission from green to blue. Images of the cells were taken with a fluorescent microscope equipped with the appropriate filter set, Excitation filter 405±10 nm, Dicroic mirror 425, Emission Filter (blue) 460±20 nm, and Emission Filter (green) 530±15 nm. Using ImageJ, an image software program provided by NIH, the amount of green and blue fluorescence was determined. To quantify the observed fusion, a fluorescence ratio was calculated by dividing the amount of blue fluorescence by the amount of green fluorescence. This value was then normalized to the non-infected control by dividing the calculated values by the average control value.

### 4.5 Papuamide A-protein binding interactions

Binding interactions were quantified by surface plasmon resonance (SPR). Studies were performed at the University of Utah Protein Interactions facility on a BIAcore 3000 instrument. Briefly, papuamide A was biotinylated using the EZ-Link Sulfo-NHS-LC-LC-Biotin reagent (Pierce Biotechnology, Rockford, IL) with reaction products purified by reverse phase high performance liquid chromatography (HPLC). Biotinylation was confirmed by mass spec and presumed to target the free amine of the 2,3-diaminobutenoic acid. Biotinylated papuamide A was captured onto a chip with a carboxymethylated dextran matrix preimmobilized with streptavidin. Analytes were then flowed over the chip in hepes buffered saline (HBS) running buffer containing 1 mg/mL bovine serum albumin to reduce nonspecific binding. Soluble CD4 (sCD4) [57] and gp120 were obtained through the AIDS Research and Reference Reagent Program, contributed by Progenics Pharmaceuticals, Inc. and Division of AIDS, NIAID, NIH, respectively. A papuamide A positive control binding peptide was identified from a library of peptides (Catalog #6405, AIDS Research and Reference Reagent Program, NIH) contributed by Division of AIDS, NIAID, NIH. Stock concentration of 5 μM for sCD4, 8.3 μM for gp120 and 500 μM for the peptide were diluted 1/100 followed by three serial dilutions in HBS for testing. Binding responses reported as an arbitrary resonance unit (RU). Data analyzed using the SCRUBBER software, developed by the University of Utah Protein Interactions Facility.

### 4.6 Pseudotype virus assay

The production of pseudotype virus was accomplished by co-transfection of 30 μg of pDHIV-3 and 15 μg envelope plasmid by calcium phosphate mediated transfection of 293FT cells. pDHIV-3 [27] is a plasmid which encodes for all HIV proteins except envelope and nef and contains a green fluorescence protein (GFP) reporter gene in place of nef. Envelope glycoprotein plasmids used included: pSCA [52], amphotropic murine leukemia virus glycoprotein (aMLV); pHCMV-VSVG [34], vesicular stomatitis virus glycoprotein (VSV); pLET-JRFL [53], a R5 tropic HIV envelope glycoprotein; and, pLET-LAI [53, 54] and pIIINL4env [50], X4 tropic HIV envelope glycoproteins.

For inhibition studies, a 1.5 mL aliquot of pseudotype virus, with or without 178 nM papuamide A, was added to 2.5 × 10^5^ CEM-SS or CEM.NKR-CCR5 cells in microfuge tube. Cells underwent a spinoculation at 1,700 × *g* for 2 hours at 25°C. Virus was aspirated and cells resuspended in 1mL RPMI, 10%FBS and plated into a 12 well culture plate. Cells were incubated for 48 hours at 37°C, 5% CO_2_ after which they were analyzed by flow cytometry. Infection rates were normalized so that the number of GFP expressing cells in the HIV infected control equaled 100.

### 4.7 Flow Cytometry

Flow cytometry was performed using a FACScan instrument (Becton Dickinson, Franklin Lakes, NJ). Cells were pelleted by centrifugation at 1,700 x *g*, washed with fluorescence-activated cell sorter (FACS) buffer (2% fetal bovine serum and 0.02% sodium azide in PBS) and resuspended in FACS buffer for analysis. The flow cytometer was set to analyze a total of 10,000 cells, gating for GFP producing cells. Analysis was performed by Cell Quest Alias software (BD Biosciences, San Jose, CA). Percent of total cells expressing GFP indicates percent cells infected. Background counts were averaged and subtracted from the infected and treated cell values.

### 4.8 Time dependent inhibition of infection

Infection of CEM-SS cells with pseudotype virus expressing IIINL4env (X4 tropic envelope) or VSV envelope was performed as described above with the exception of 0.5 mL of virus added with 1 mL of fresh medium. This allows for consistency between treatments because medium does not have to be changed after spinoculation. Initial time-dependency experiments were performed with IIINL4env pseudotype virus. Papuamide A (178 nM), AZT (3.7 μM) or C34 (233 nM) was added at 0, 1 and 2 hours after virus addition. To determine effect of cell pretreatment, cells were treated with papuamide A overnight, pelleted by centrifugation at 1,700 × *g* and papuamide A containing medium aspirated. A second experiment with delayed addition of papuamide A to VSV and IIINL4env pseudotype virus was also performed.

### 4.9 Assessment of papuamide A preincubation on viral fusion

HeLa T4 cells were infected with NL4-3 BlaM virus and treated with 710 nM papuamide A, following these three protocols: Virus pretreated with papuamide A for 30 minutes at room temperature prior to dilution and addition to the cells; Cells pretreated with papuamide A for 2 hours followed by aspiration of papuamide A containing medium and addition of virus; Virus and papuamide A added to the cells at the same time.

### 4.10 Effect of VSV pseudotype virus pretreatment

VSV pseudotype virus was incubated with or without 178 nM papuamide A overnight in a 37°C, 5% CO_2_ incubator. Virus was centrifuged at 25,000 × g for 2 hours at 4°C to pellet the virus. Virus was resuspended in fresh RPMI, 10% FBS medium. Non-pretreated virus, non-pretreated virus plus 178 nM papuamide A and virus pretreated with 178 nM papuamide A were then used to infect CEM-SS cells by spinoculation and infectivity determined by flow cytometry after 24 hours.

### 4.11 Analysis

Image J (NIH) was used to quantify fluorescence from microscope images. Prism software (GraphPad Software) was used to generate and analyze dose response graph. Statistical analysis was performed using the paired Student *t*-test with a significance level at p < 0.05.

## Figures and Tables

**Figure 2a.**
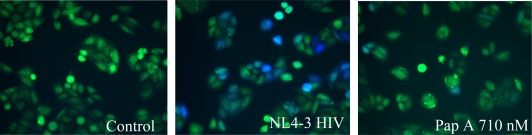
Representative fluorescence microscope images of the virion based fusion assay. Panels show uninfected green cells (Control), the presence of infected blue cells (NL4-3 HIV) and a reduction of infected blue cells in the presence of a viral entry inhibitor (Pap A).

**Figure 2b.**
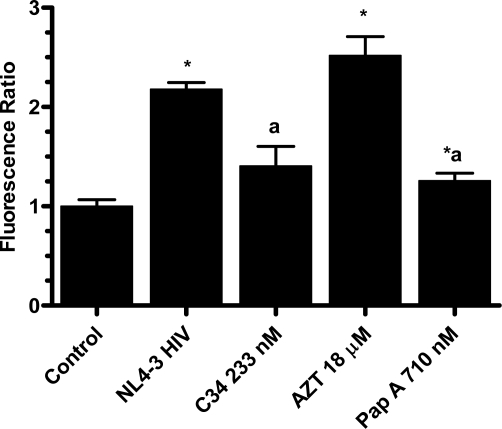
Inhibition of NL4-3 (X4 tropic virus) entry in the presence of papuamide A (Pap A), the known fusion inhibitor C34 and reverse transcriptase inhibitor azidothymidine (AZT) at final concentrations of 710 nM, 233 nM and 18 μM, respectively. Control represents uninfected cells and NL4-3 HIV represents untreated infected cells. Viral entry is reported as a fluorescence ratio calculated from the amount of blue versus green fluorescence, normalized to control. Results graphed as the mean ± standard error, n≥4. Fluorescence ratio was significantly different (p < 0.05) from control (*) or from untreated HIV infected cells (a).

**Figure 2c.**
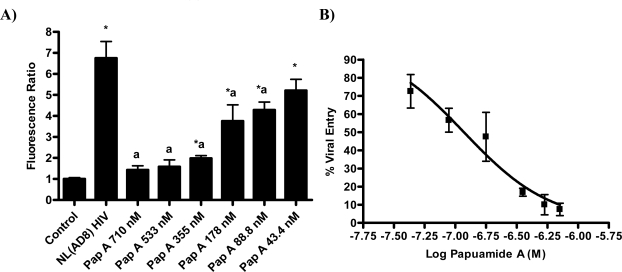
Dose dependent inhibition of NL4(AD8) (R5 tropic virus) entry by papuamide A. Papuamide A was tested at concentrations of 710, 533, 355, 178, 88.8 and 43.4 nM. Panel A shows the dose dependent inhibition of viral entry and panel B is a dose response curve generated from the same data normalized to indicate percent viral entry. Data is representative of experiments with similar results repeated twice and is graphed as the mean ± standard error, n≥3. Fluorescence ratio was significantly different (p < 0.05) from control (*) or from untreated HIV infected cells (a).

**Figure 5a.**
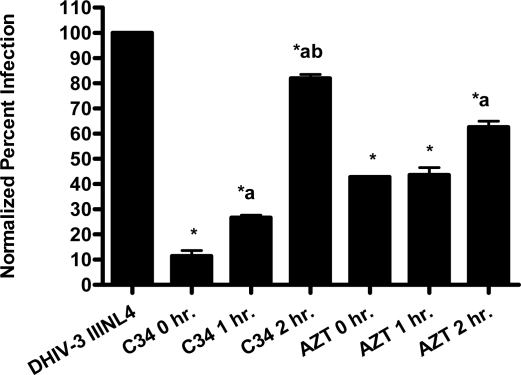
Time dependent inhibition of infection by controls C34 (233 nM) and AZT (3.7 μM). CEM-SS cells were infected with DHIV-3 pseudotype virus expressing an X4 tropic envelope (IIINL4) and treated with control drugs at the stated time points. DHIV-3 represents infected cells treated with vehicle. Data is graphed as average with range from duplicate determinations. Percent infection was significantly different (p < 0.05) between infected cells that were untreated and those treated (*), between 0 hour and other timepoint treatments (a), and between 1hour and 2 hour timepoint treatments (b).

**Figure 5b.**
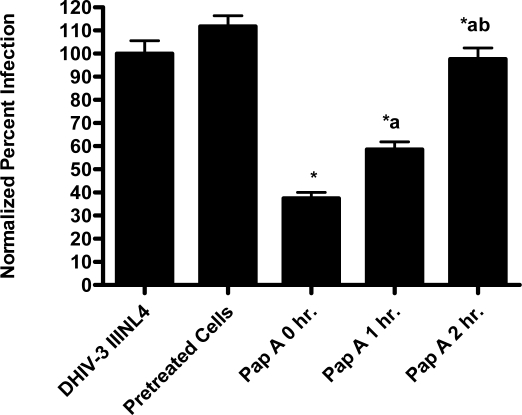
Time dependent inhibition of infection by papuamide A. CEM-SS cells were infected with DHIV-3 pseudotype virus expressing IIINL4 envelope and treated with papuamide A (178 nM) at the stated time points. DHIV-3 represents infected cells treated with vehicle. Data is graphed as the mean ± standard error, n≥3. Percent infection was significantly different (p < 0.05) between infected cells that were untreated or treated with papuamide A (*), between 0 hour and other timepoint treatments (a), and between 1hour and 2 hour timepoint treatments (b).

**Figure 5c.**
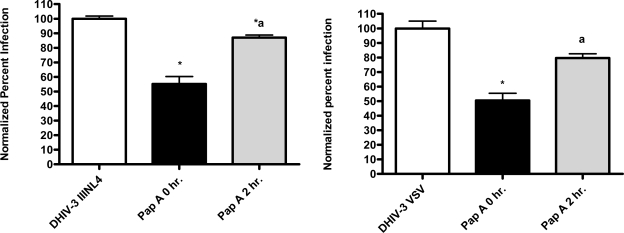
Time dependent inhibition of IIINL4env versus VSV-G pseudotype virus by papuamide A. Papuamide A, 178 nM, was added to IIINL4env and VSV-G pseudotype virus at the time of infection, 0 hr., (black bars) and two hours (grey bars) after virus addition tested alongside untreated infected cells (white bars). Data is graphed as the mean ± standard error, n≥3 for virus pseudotyped with HIV IIIenv and as average with error bars from duplicate determinations for virus pseudotyped with VSV envelope.

**Figure 6a.**
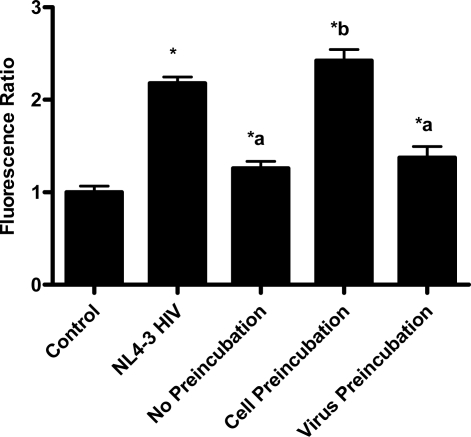
Papuamide A does not inactivate the cell to inhibit viral entry. Inhibition of NL4-3 BlaM-Vpr virus entry was determined when 178 nM papuamide A was preincubated with target cells followed by washing, preincubated with virus followed by dilution, and no preincubation (added at the same time as virus). Control represents uninfected cells and NL4-3 HIV represents untreated infected cells. Viral entry reported as a fluorescence ratio. Data is graphed as the mean ± standard error, n≥3. Response ratio was significantly different (p < 0.05) from control (*), between HIV infected cells that were untreated or treated with papuamide A (a) and between papuamide A cells treated at time of virus addition (no preincubation) versus other papuamide A treatment protocols (b).

**Figure 6b.**
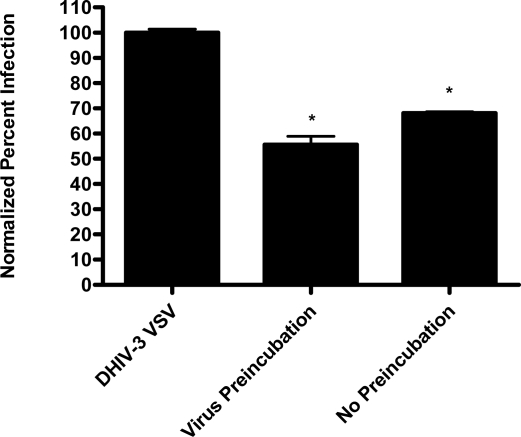
Papuamide A interacts directly with the virus. VSV pseudotype virus was incubated overnight with or without 178 nM papuamide A. Non-preincubated virus, preincubated virus, and non-preincubated virus with papuamide A were used to infect CEM-SS cells. Infection was determined by quantifying the percent of total cells expressing GFP. Data is representative of experiments with similar results repeated at least twice and is graphed as the mean ± standard error, n≥3. Percent infection was significantly different (p < 0.05) between infected cells that were untreated or treated with papuamide A (*).

**Figure 7a.**
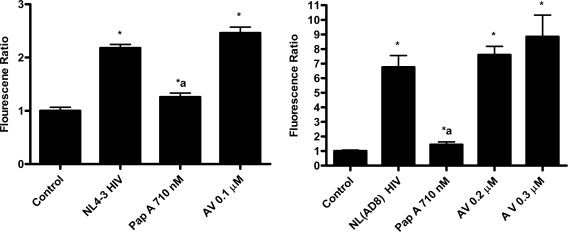
Binding of phosphatidylserine alone in not sufficient enough to inhibit viral entry. Binding of phosphatidylserine by annexin V (AV) was assessed in the virion fusion assay against X4 (NL4-3) and R5 (NL(AD8)) tropic virus. Control represents uninfected cells and NL4-3 or NL(AD8) HIV represents untreated infected cells. Viral entry is reported as a fluorescence ratio. Data is graphed as the mean ± standard error, n≥4. Response ratio was significantly different (p < 0.05) from control (*), or between HIV infected cells that were untreated or treated (a).

**Figure 7b.**
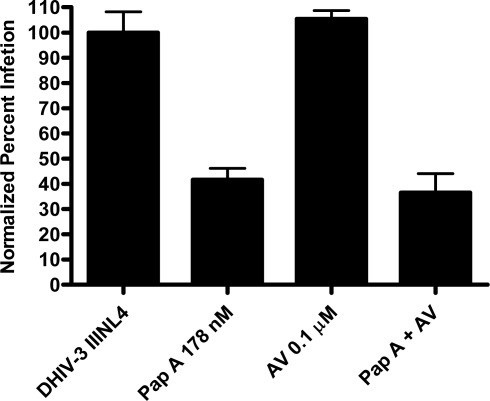
Co-treatment of papuamide A with AV does not affect papuamide A’s inhibition of infection by HIV envelope pseudotype virus. CEM-SS cells were infected with DHIV-3 pseudotype virus expressing IIINL4 envelope and treated with papuamide A (Pap A, 178 nM), annexin V (AV, 0.1 μM), or papuamide A and annexin V. DHIV-3 represents infected cells treated with vehicle. Data is graphed as the mean ± standard error, n=3.

**Table 1.** Values for SPR data analysis.

	Analyte		
	sCD4	gp120	Peptide
Molecular Weight	26,000	120,000	1898
Expected Response at Saturation (RU)	9,743	44,968	712
Observed Response (RU)at Maximum Analyte Concentration	10	35	30
Fold Difference of Responses	974	1284	24
K_D_ (μM)	0.08	2	176
Maximum Analyte Concentration Tested (μM)	0.05	0.08	5
Fold Difference between K_D_ and Analyte Concentration	1.6	24	35
Expected Response at Maximum Concentration Tested (RU)	6089	1874	20

Binding interactions between papuamide A and the above listed analytes were measured using surface plasmon resonance (SPR). Above defined values were used for data analysis calculations. Formulas provided in text.
